# Characterization of heterosis and genomic prediction‐based establishment of heterotic patterns for developing better hybrids in pigeonpea

**DOI:** 10.1002/tpg2.20125

**Published:** 2021-08-01

**Authors:** Rachit K. Saxena, Yong Jiang, Aamir W Khan, Yusheng Zhao, Vikas Kumar Singh, Abhishek Bohra, Muniswamy Sonappa, Abhishek Rathore, C.V. Sameer Kumar, Kulbhushan Saxena, Jochen Reif, Rajeev K. Varshney

**Affiliations:** ^1^ International Crops Research Institute for the Semi‐Arid Tropics (ICRISAT) Patancheru Telangana 502324 India; ^2^ Leibniz‐Institute of Plant Genetics and Crop Plant Research (IPK) Corrensstr. 3, D‐06466 Stadt Seeland Germany; ^3^ Indian Council of Agricultural Research – Indian Institute of Pulses Research Kanpur 208024 India; ^4^ Zonal Agricultural Research Station, Univ. of Agricultural Sciences – Raichur Gulbarga Karnataka 585101 India; ^5^ Professor Jayashankar Telangana State Agricultural Univ. Rajendranagar Hyderabad Telangana 500030 India; ^6^ Al Mudaredh, Al Ain, Abu Dhabi, United Arab Emirates; ^7^ State Agricultural Biotechnology Centre, Centre for Crop and Food Innovation, Food Futures Institute Murdoch Univ. Murdoch WA 6150 Australia

## Abstract

Whole‐genome resequencing (WGRS) of 396 lines, consisting of 104 hybrid parental lines and 292 germplasm lines, were used to study the molecular basis of mid‐parent heterosis (MPH) and to identify complementary heterotic patterns in pigeonpea [*Cajanus cajan* (L.) Millsp.] hybrids. The lines and hybrids were assessed for yield and yield‐related traits in multiple environments. Our analysis showed positive MPH values in 78.6% of hybrids, confirming the potential of hybrid breeding in pigeonpea. By using genome‐wide prediction and association mapping approaches, we identified 129 single nucleotide polymorphisms and 52 copy number variations with significant heterotic effects and also established a high‐yielding heterotic pattern in pigeonpea. In summary, our study highlights the role of WGRS data in the study and use of heterosis in crops where hybrid breeding is expected to boost selection gain in order to ensure global food security.

## Core Ideas

- We characterized the molecular basis of mid‐parent heterosis.
- We established the molecular foundation of genome‐wide prediction for breeding hybrids.
- We identified the loci contributing to mid‐parent heterosis.
- We predicted the best possible combinations for generating high‐yielding cultivars.

**Abbreviations**
A‐lines: cytoplasmic male‐sterile lines
B‐lines: maintainer lines
BLUE: best linear unbiased estimations
CMS: cytoplasmic male sterile
CNV: copy number variation
F_ST_
: fixation index
GWAS: genome‐wide association study
MPH: mid‐parent heterosis
QTL: quantitative trait locus
R‐lines: restorer lines
SNP: single nucleotide polymorphism
WGRS: whole‐genome resequencing.

## 1 INTRODUCTION

Plant‐based proteins are the best solution for providing cheap and high‐quality protein. Pulses are very promising in this context; among these, pigeonpea [*Cajanus cajan* (L.) Millsp.] with 20% protein content, is a greatly valued crop, as it can grow well under diverse climatic conditions and require fewer inputs. The productivity of pulses, especially pigeonpea, has been stagnating for many decades. At present, pigeonpea suffers from a low yield plateau, and there is a need to enhance the productivity of this crop. The development and promotion of high‐yielding hybrids appears to be a game‐changing step in the right direction. With this objective, an efficient hybrid breeding technology with an on‐farm yield advantage of 30 to 40% has been developed in pigeonpea (Saxena et al., 2018). This breakthrough has made pigeonpea a unique pulse and legume crop where commercial hybrids have become available. The success of hybrid pigeonpea breeding will depend on an understanding of the mechanism underlying the process of heterosis and its extensive utilization through clustering suitable germplasm into genetically complementary heterotic groups.

Heterosis is the phenomenon of the first filial (F_1_) generation outperforming their homozygous parental lines, which is widely used in plant breeding. The specific definition of heterosis varies depending on the benchmark of the parental performance used for the comparison. As the F_1_ hybrid inherits half of its genome from each parent, it is plausible to study the mid‐parent heterosis (MPH), defined as the difference between the genetic values of the hybrid and the average of the parents. Though the phenomenon of heterosis at the molecular level has been studied in the past, but this has been restricted to either the model plant *Arabidopsis thaliana* (L.) Heynh. (Kim et al., 2002) or three cereal crops, namely rice (*Oryza sativa* L.) (He et al., 2010), maize (*Zea mays* L.) (He et al., 2013), and wheat (*Triticum aestivum* L.) (Jiang et al., 2017), as well as some vegetable crops like tomato (*Solanum lycopersicum* L.) (Krieger et al., 2010). Despite of all this effort and experimental validation, the mechanism(s) of the molecular basis of heterosis is poorly understood. Nevertheless, complementary heterotic groups have been defined in maize and other crops for better exploitation of heterosis to generate improved parental lines and hybrids (Fan et al., 2009). In most of these crops, heterotic groups were defined by testing hybrid combinations of parental lines in field evaluations. However, evaluating all the possible hybrid combinations in larger set of parental lines in the field, especially for crops with narrow genetic diversity, is not possible. Therefore, complete information on combining ability of potential parental lines in a hybrid breeding program cannot be generated. To address these issues, a number of approaches, including molecular marker‐based genetic distance (Melchinger, 1999), identification of genome‐wide superior alleles (Springer & Stupar, 2007), heterotic quantitative trait loci (QTLs) (Lippman & Zamir, 2007), molecular heterozygosity and hybrid performance (Reif et al., 2003), and metabolite‐based predictions (Riedelsheimer et al., 2012) have been used. Furthermore, to maximize the short‐ and long‐term selection gains in wheat, a simulated annealing algorithm based on genome‐wide prediction has been established for defining heterotic groups (Zhao et al., 2015). However, in the legumes, which play an important role in food and nutrition security as well as environmental sustainability, none of the above approaches have been used.

In order to understand the molecular basis of heterosis for grain yield and to establish a promising heterotic pattern in pigeonpea hybrids, we conducted the present study. We used multiyear and multilocation phenotyping data from 104 parental lines and 435 of their single‐cross hybrid progeny, combined with 292 lines from a previous study (Varshney et al., 2017). Whole‐genome resequencing (WGRS) data for all 396 inbred lines were used to identify loci contributing to MPH and to define the heterotic groups.

**Core Ideas**

- We characterized the molecular basis of mid‐parent heterosis.
- We established the molecular foundation of genome‐wide prediction for breeding hybrids.
- We identified the loci contributing to mid‐parent heterosis.
- We predicted the best possible combinations for generating high‐yielding cultivars.

## 2 MATERIALS AND METHODS

### 2.1 Plant materials and field evaluation

The plant material used in this study consisted of 738 single‐cross hybrids derived from 104 parental lines (Supplemental Table S1). However, according to the seed availability, 435 unique single‐cross hybrids generated by crossing 56 restorer lines (R‐lines; males) with nine cytoplasmic male‐sterile lines (A‐lines; female) could be used in field evaluations. In total, 104 parental lines, 435 unique single‐cross hybrids, and 14 checks were tested in two environments. Field trials were conducted in two replications in alpha lattice designs with 51 blocks and a block size of 10 plots. In addition, 292 lines from 304 inbred lines evaluated in previous study (Varshney et al., 2017) were also included in this study. In brief, the phenotyping data were collected from 2 yr in two locations (International Crops Research Institute for the Semi‐Arid Tropics and Gulbarga) in India. The 304 inbred lines were tested in each location with two replications in alpha lattice designs with eight blocks and a block size of 38 plots. Three individual plants from each genotype (inbred lines and hybrids) in every replication were used to collect the trait phenotyping data for nine agronomic traits including grain yield per plant (g per plant), days to 50% flowering, days to 75% maturity, plant height (cm), number of pods per plant, 100‐seed weight (g), number of seeds per pod, number of primary branches per plant, and number of secondary branches per plant. The phenotyping procedure and scoring standard followed the practices outlined in the Genebank manual (Upadhyaya & Gowda, 2009).

### 2.2 DNA extraction and sequencing

In the present study, single plants from each of the 104 parental lines were used to collect young leaves and total DNA was extracted with the cetyltrimethylammonium bromide method following standard procedure. Paired‐end sequencing libraries with an insert size of approximately 400 bp were constructed using genomic DNA and sequenced on an Illumina HiSeq 2000 sequencer. Resequencing reads were mapped on to the pigeonpea reference genome (Varshney et al., 2012) by BWA (Version 0.5.9) (Heng & Durbin, 2009) with the default parameters. Mapped reads were converted into BAM files by SAMtools (Version 0.1.18) (Li et al., 2009) and duplicated reads were removed. The genome coverage of the mapped reads on the reference genome was calculated with GATK (Version 1.4‐11) (McKenna et al., 2010).

### 2.3 Sequence variations and annotation

BCFtools (Version 0.1.17) (Heng & Durbin, 2009) in SAMtools was used to detect sequence variations including single nucleotide polymorphisms (SNPs) and indels in 104 parental lines. Identified sequence variations were annotated with annovar (Version 2011Nov28) (Wang & Hakonarson, 2010) and SnpEff (Version 3.2) (Cingolani et al., 2012). Further, the SNPs were counted with VCFtools (Version 0.1.10) (Danecek & Auton, 2011") and the indels counted with bedops (Version 2.4.3) (Neph et al., 2012) and in‐house perl scripts. Furthermore, combined sequencing data from 396 lines including previously published sequence data on 292 inbred lines (Varshney et al., 2017) and 104 parental lines were also used for sequence variation analysis, as mentioned above.

### 2.4 Analyses of phenotyping data across environments

#### 2.4.1 Inbred lines

We performed a combined analysis for the 104 parental lines generated in this study and the 304 lines evaluated previously. The following linear mixed model was used to estimate the variance components and the best linear unbiased estimations (BLUEs) across environments (an environment is the combination of a location and a year):

(1) Y∼G+E+G:E+R,

where *Y* is the yield, *G* is the genotype, *E* is the environment, and *R* is the residual effect.

To estimate the BLUEs, the genotype effects were treated as fixed effects and the remaining effects, including the residuals, were treated as random. To estimate the variance components, all effects were treated as random. The broad‐sense heritability was then calculated as the ratio of genotypic to phenotypic variance:

(2) $$h^{2}=\;\frac{\sigma_{G}^{2}}{\sigma_{G}^{2}+\frac{\sigma_{G\times E}^{2}}{N_{E}}+\frac{\sigma_{E}^{2}}{N_{E}\times N_{R}}},$$

where $N_{E}$ refers to the number of environments,$\;N_{R}$ is the average number of replications per location. $\sigma_{G}^{2}$ is the genetic variance, $\sigma_{G\times E}^{2}$ is the variance of the genotype × environment interaction, and $\sigma_{E}^{2}$ refers to the variance of the residuals.

#### 2.4.2 Hybrids

The phenotypic data were analyzed via a one‐step approach assuming heterogeneous variance of the residuals for each location. We used the following linear mixed model to estimate the BLUEs of the genotypes across the locations:

(3) Y∼G+E+G:E+E:Rep+E:Rep:B+R,

where *Y* is the yield, *G* is the genotype, *E* is the environment, Rep is the replicate, *B* is the block, and *R* is the residual. Genotype was treated as a fixed effect and the others were random effects. The distributions of the BLUEs for these traits revealed that hybrids had higher yield and earlier maturity than their parental inbred lines. The genetic variance was further decomposed into the variance of the general combining ability effects of male $\left(\sigma_{GCA_{m}}^{2}\right)$ and female lines ($\sigma_{GCA_{f}}^{2})$ and specific combining ability effects ($\sigma_{SCA}^{2}$) by the following linear mixed model:

(4) $$\begin{array}{ccc} Y & ∼ & Gr+E+M+F+M:F+E:\mathrm{Rep}+E\\ & & :\mathrm{Rep}:\;B+R, \end{array}$$

where *Y* is the yield, *Gr* is the group, *E* is the environment, *M* is the male lines, *F* is the female lines, Rep is the replicate, *B* is the block and *R* is the residual effect. Group refers to a fixed effect for the check, male, female, and hybrids. All other effects were treated as random effects with heterogeneous error variance in each environment. Broad‐sense heritability was calculated as the ratio of genotypic to phenotypic variance.

### 2.5 Applying a quantitative genetic framework to study the genetic basis of heterosis

The phenotyping and WGRS data were combined to investigate the genetic architecture of MPH for grain yield. Hybrids with missing phenotyping data were filtered out and 62 parental lines and 378 hybrid progeny remained. After quality control for missing values (<5%), minor allele frequency (>5%), and heterozygosity (<2.5%), 128,067 high quality SNPs were considered in the analyses. We applied the quantitative genetic framework developed by Jiang et al. (2017) to study the genetic basis of MPH. Below, we briefly describe the approach. For more details, we refer to the reader Jiang et al. (2017).

#### 2.5.1 Mid‐parent heterosis for grain yield

The MPH was defined as:

(5) $$MPH=F_{1}-\frac{1}{2}\left(P_{m}+P_{f}\right),$$

where $F_{1}$ denotes the value of a hybrid, and $P_{m}$ and $P_{f}$ denote the values of the male and female parent, respectively.

#### 2.5.2 Partitioning of genetic variance components for MPH

Genetic variance components for MPH were estimated by fitting an extended genomic best linear unbiased prediction model including dominance and digenic epistatic effects (Jiang & Reif, 2015; Xu et al., 2014). Briefly, the model can be described as follows:

(6) $$y\;=g_{d}\;+g_{aa}+g_{ad}+g_{dd}+e,$$

In this model, y is the vector of the MPH values for all hybrids; genetic values are represented as dominance$\;(g_{d})$, additive‐by‐additive ($g_{aa}$), additive‐by‐dominance ($g_{ad}$), and dominance‐by‐dominance ($g_{dd}$) effects; and e is a residual term. In the model, we assumed that $g_{d}∼N\left(0,K_{d}\sigma_{d}^{2}\right)$, $g_{aa}∼N\left(0,K_{aa}\sigma_{aa}^{2}\right)$, $g_{ad}∼N\left(0,K_{ad}\sigma_{ad}^{2}\right)$, $g_{dd}∼N\left(0,K_{dd}\sigma_{dd}^{2}\right)$, and $e∼N(0,TT^{\prime }\sigma_{e}^{2})$, where $K_{d}$, $K_{aa}$, $K_{ad}$, and $K_{dd}$ are marker‐derived kinship matrices for the different genetic effects. T is a r×(r+s) matrix of linear transformation from the vectors of the original trait (grain yield) to the vectors of MPH, where r is the number of hybrids and s is the number of parental lines. The reason why the residual term was not independently distributed is that we assumed independent residual terms for the original trait, but the MPH values were derived from the original trait values in the form of the linear transformation T. The marker‐derived kinship matrices are also specific to MPH instead of the original trait. The variance components $\sigma_{d}^{2}$, $\sigma_{aa}^{2}$, $\sigma_{ad}^{2}$, and $\sigma_{dd}^{2}$ were estimated by the multi‐kernel method in the R package BGLR (Pérez & De Los Campos, 2014).

#### 2.5.3 Definition of heterotic effects

The heterotic effect of a locus is the genetic contribution of the locus to MPH, which is a complex combination of the dominance effect of the locus itself and the epistatic interaction effects with the entire genetic background (Jiang et al., 2017). The precise definition is as follows:

Let Q be the set of all QTL for the original trait. Quantitative trait loci were coded as 0, 1, or 2, depending on the number of chosen alleles at each locus. Considering one hybrid, we use $R_{kl}$ (k,l=0 or 2) to denote the subset of loci where the female parent has Genotype k and the male parent has Genotype l. For i,j∈Q, $d_{i}$ is the dominance effect of the i ^th^ QTL, $aa_{ij}$ is the additive‐by‐additive epistatic effect between the i ^th^ and the j ^th^ QTL, $ad_{ij}$ is the additive‐by‐dominance epistatic effect between the i ^th^ and the j ^th^ QTL, and $dd_{ij}$ is the dominance‐by‐dominance epistatic effect between the i ^th^ and thej‐ QTL. The heterotic effect of the i ^th^ locus was defined as:

(7) $$h_{i}=\;\;\left\{\begin{array}{c} d_{i}-\frac{1}{2}\sum_{j\in R_{20}}aa_{ij}+\frac{1}{2}\sum_{j\in R_{02}}aa_{ij}+\frac{1}{2}\sum_{j\in R_{22}\;}ad_{ji}\\ -\;\frac{1}{2}\sum_{j\in R_{00}\;}ad_{ji}+\frac{1}{2}\sum_{j\in R_{20}\cup R_{02}}dd_{ij}\;\;\;\;\;\;\;\;\;\;\;\;\;\;\;\;\;\;if\;i\in R_{20}\\ d_{i}-\frac{1}{2}\sum_{j\in R_{02}}aa_{ij}+\frac{1}{2}\sum_{j\in R_{20}}aa_{ij}+\frac{1}{2}\sum_{j\in R_{22}\;}ad_{ji}\\ -\;\frac{1}{2}\sum_{j\in R_{00}\;}ad_{ji}+\frac{1}{2}\sum_{j\in R_{20}\cup R_{02}}dd_{ij}\;\;\;\;\;\;\;\;\;\;\;\;\;\;\;\;\;\;if\;i\in R_{02}\\ \frac{1}{2}\sum_{j\in R_{20}\cup R_{02}\;}ad_{ij}\;\;\;\;\;\;\;\;\;\;\;\;\;\;\;\;\;\;\;\;\;\;\;\;\;\;\;\;\;\;\;\;\;\;\;\;\;\;\;\;\;\;\;\;\;\;\;\;\;\;\;\;\;\;\;\;\;\;\;\;\;\mathrm{if}\;i\in R_{22}\\ -\;\frac{1}{2}\sum_{j\in R_{20}\cup R_{02}\;}ad_{ij}\;\;\;\;\;\;\;\;\;\;\;\;\;\;\;\;\;\;\;\;\;\;\;\;\;\;\;\;\;\;\;\;\;\;\;\;\;\;\;\;\;\;\;\;\;\;\;\;\;\;\;\;\;\;\mathrm{if}\;i\in R_{00} \end{array}\right..$$

With this definition, the MPH value of each hybrid is the sum of heterotic effects across all loci (i.e., $MPH\;=\sum_{i\in Q}h_{i}\;$).

#### 2.5.4 Genome‐wide scan for significant heterotic effects

We applied the following three‐step procedure to detect significant heterotic effects: First, a genome‐wide association study (GWAS) was performed to identify significant dominance and digenic epistatic effects. We used a standard linear mixed model with a marker‐derived kinship matrix controlling for the structure of multiple levels of relatedness and polygenic background effects (Yu et al., 2006). Since the presence of epistasis was assumed, it was necessary to apply a model controlling the polygenic background effects, which consisted of both main and epistatic effects (Xu, 2013). The model can be described as follows:

(8) $$y\;=\;m\alpha +g_{d}+g_{aa}+g_{ad}+g_{dd}+e,$$

where y, $g_{d}$, $g_{aa}$, $g_{ad}$, $g_{dd}$, and e are the same as in Equation 6. In particular, α is the genetic effect being tested and m is the corresponding coefficient. More precisely, α is the dominance effect of any marker or the epistatic interaction effect for any pair of markers. We assumed that α is an unknown fixed parameter. The other assumptions are the same as in Equation 6. In the second step, the significant component effects were integrated into the heterotic effects. All nonsignificant effects were set to zero. In order to reduce any possible upward bias from accumulating the GWAS‐estimated effects for highly correlated marker pairs, we re‐estimated the significant effects with the Bayesian ridge regression model before integrating the effects. Finally, the heterotic effect ($h_{i}$) of each locus was tested by a permutation test. More precisely, for each locus, the MPH values of all the hybrids can be predicted from the heterotic effect of this particular locus. Next, the Pearson correlation coefficient between the predicted and observed MPH values was calculated and a permutation test for the correlation coefficient was performed.

#### 2.5.5 Estimating the number of independent markers and marker pairs

To investigate the number of independent markers for all significant markers detected by GWAS, we applied a principal component analysis to the matrix of significant markers and sought for the minimal number of PCs that explained 99% of the variance. For marker pairs that showed significant epistatic effects, we first produced pseudomarkers derived from the scores of the two markers, then applied this approach to the matrix of pseudomarkers.

#### 2.5.6 Testing the heterotic effects of copy number variations

At each site where a copy number variation (CNV) was detected, the CNV was treated as a multiallelic marker (i.e., each possible copy number at the site was considered as a specific allele). In particular, we did not assume linear effects with respect to the number of copies. To test the contribution of CNVs to heterosis, we applied the multiallelic version of the quantitative genetic framework (Jiang et al., 2017); the model was the same as the haplotype‐based model described therein. We considered only the dominance effects of the CNVs, meaning the interaction effects among different alleles within each CNV. The interaction effects across CNVs were not included in the model because the power of association test would be severely impaired by the extremely low frequencies of multilocus, multiallele genotypes.

### 2.6 Genome‐wide prediction for hybrid performance

We applied the ridge regression best linear unbiased prediction model that included additive and dominance effects. The general form of the model is as follows:

(9) $$y\;=1_{n}\;\mu +Z_{A}a+Z_{D}d+e,$$

where *1_n_* is a vector of ones and n is the number of lines; *μ* refers to the overall mean across environments; *Z_A_* is a design matrix of size *n × m* for the additive effect of the markers, where m refers to the number of markers and the elements of *Z_A_* are coded as −1, 0, and 1; $a\;={\left(a_{1},\;a_{2},\ldots ,a_{m}\right)}^{T}\;$ is a vector of lengthm, where $a_{i}$ denotes the additive effect for the *i* ^th^ marker; *Z_D_* is a matrix with a *n × m* design for the dominance effect of the markers in which the elements of *Z_D_* are coded as 0 and 1; $d\;={\left(d_{1},\;d_{2},\ldots ,d_{m}\right)}^{T}\;$is a vector of lengthm, where$\;d_{i}$ denotes the dominance effect for the *i* ^th^ marker; and $e\;={\left(e_{1},\;e_{2},\ldots ,e_{n}\right)}^{T}\;\;$is a vector of length *n* and, where $e_{j}$ is the residual for the *j* ^th^ line.

We evaluated the ability to predict grain yield via cross‐validations with different relatedness between the training and test sets. The training set comprised the 292 inbred lines and a subset of hybrid parental lines as well as some of their hybrid progeny. More precisely, we randomly sampled seven (out of nine) female and 35 (out of 56) male parental lines as well as 160 hybrids derived from them. From the remaining hybrids, test sets with three successively decreasing degrees of relatedness to the training sets were formed. Test set T2, which was most closely related to the training set, included only hybrids derived from the same parents as the hybrids in the training set, whereas the less related test set (T1) included hybrids sharing one parent with the hybrids in the training set and the least related test set (T0) included only hybrids that had no parents in common with the training set. This sampling process was repeated 100 times, and the number of hybrids in the test sets ranged from 61 to 87, 176 to 198, and 41 to 54 for the T2, T1, and T0 populations, respectively. For each sampling round, we estimated the marker effects in the training set, which was then used to predict the performance of the hybrids in the T2, T1, and T0 test sets. The prediction accuracy for each test set was estimated as the Pearson correlation coefficient between the predicted and the observed hybrid performance divided by the square root of the heritability.

### 2.7 Establishing high‐yielding heterotic groups

Establishment of the heterotic groups can be briefly described as follows: First, we predicted the yield performance of all possible 78,210 single‐cross hybrids that could be derived from the 396 inbred lines (104 parental lines + 296 additional inbred lines) with the 396 lines and 435 hybrids used as the training population and the same model as mentioned in Equation 9 in the previous subsection. Based on the predicted performance of all 78,210 hybrids, the simulated annealing algorithm (Zhao et al., 2015) was implemented to search for promising heterotic groups. The group size was set to be 20 lines for each group.

## 3 RESULTS

### 3.1 Boosting grain yield through hybrid breeding in pigeonpea

A hybrid pigeonpea population consisting of 435 single‐cross hybrids was generated in a partial factorial design that crossed 104 parental lines including nine cytoplasmic male sterile (CMS) or A‐lines, 13 maintainer (B‐lines), and 82 R‐lines (Supplemental Table S1). Replicated field trials for all hybrids and their parental lines were conducted at two locations in India. The heritability estimates amounted to 0.9 for grain yield (Supplemental Table S2), indicating the high quality of the phenotypic data. Hybrids outyielded their parental lines (Supplemental Figure S1) and MPH, defined as the difference between the performance of the hybrid and the average of its parents, averaged 28.1 g per plant (Supplemental Figure S2). In total, 297 hybrids (78.6%) showed positive MPH values, which were in accordance with estimates from a historical dataset consisting of 68 hybrids derived from 44 parental lines (see the the models in Section 2.4). Thus, analysis of the phenotypic data supported the potential to increase grain yield through hybrid pigeonpea breeding.

### 3.2 Whole genome resequencing (WGRS) of hybrid parental lines and sequence variations

High‐quality WGRS data were generated for the 104 parental lines. We generated 5.2 billion paired‐end reads with an average read length of 247.5 bp (∼511 Gb of sequence) that were mapped to the reference genome of the pigeonpea cultivar ‘Asha’ (ICPL 87119) (Varshney et al., 2012) by BWA (Heng & Durbin, 2009). We obtained sequencing depths in the range of 5× to 8× and genome coverage of approximately 87% (Supplemental Table S1). The WGRS data provided >4.4 million variants across the 104 hybrid parental lines (Table 1, Supplemental Table S3). This included ∼4.0 million SNPs and ∼0.4 million small indels of 1–5 bp. Maintainer lines possessed higher levels of sequence variation (11.54% higher for SNPs and 11.23% higher for the indels) than the CMS lines (Supplemental Table S4, Supplemental Table S5), whereas among the restorer lines, the highest level of sequence variation was presented (Supplemental Table S6).

**TABLE 1:** Summary of whole‐genome variations identified in 104 parental lines of the hybrids and across the combined set of 396 lines

**Pseudomolecule**	**104 lines**			**396 lines**		
	**SNPs** ^a^	**Indels**	**Total variations**	**SNPs**	**Indels**	**Total variations**
CcLG01	125,893	17,553	143,446	625,963	79,467	705,430
CcLG02	191,125	28,245	219,370	1,270,071	164,783	1,434,854
CcLG03	129,801	21,154	150,955	993,087	133,761	1,126,848
CcLG04	72,296	11,820	84,116	425,536	57,428	482,964
CcLG05	27,746	4,080	31,826	169,317	22,927	192,244
CcLG06	123,763	19,307	143,070	803,139	107,726	910,865
CcLG07	108,416	15,710	124,126	670,655	83,287	753,942
CcLG08	109,599	16,410	126,009	695,495	88,069	783,564
CcLG09	70,914	9,451	80,365	366,769	45,291	412,060
CcLG10	137,565	16,878	154,443	775,357	88,675	864,032
CcLG11	274,166	36,674	310,840	1,659,785	199,570	1,859,355
CcLG00	2,642,085	259,592	2,901,677	11,196,947	1,113,407	12,310,354
Total	4,013,369	456,874	4,470,243	19,652,121	2,184,391	21,836,512

^a^ SNPs, single nucleotide polymorphisms; CcLG, *Cajanus cajan* linkage group or pseudomolecule.

**FIGURE 1:**
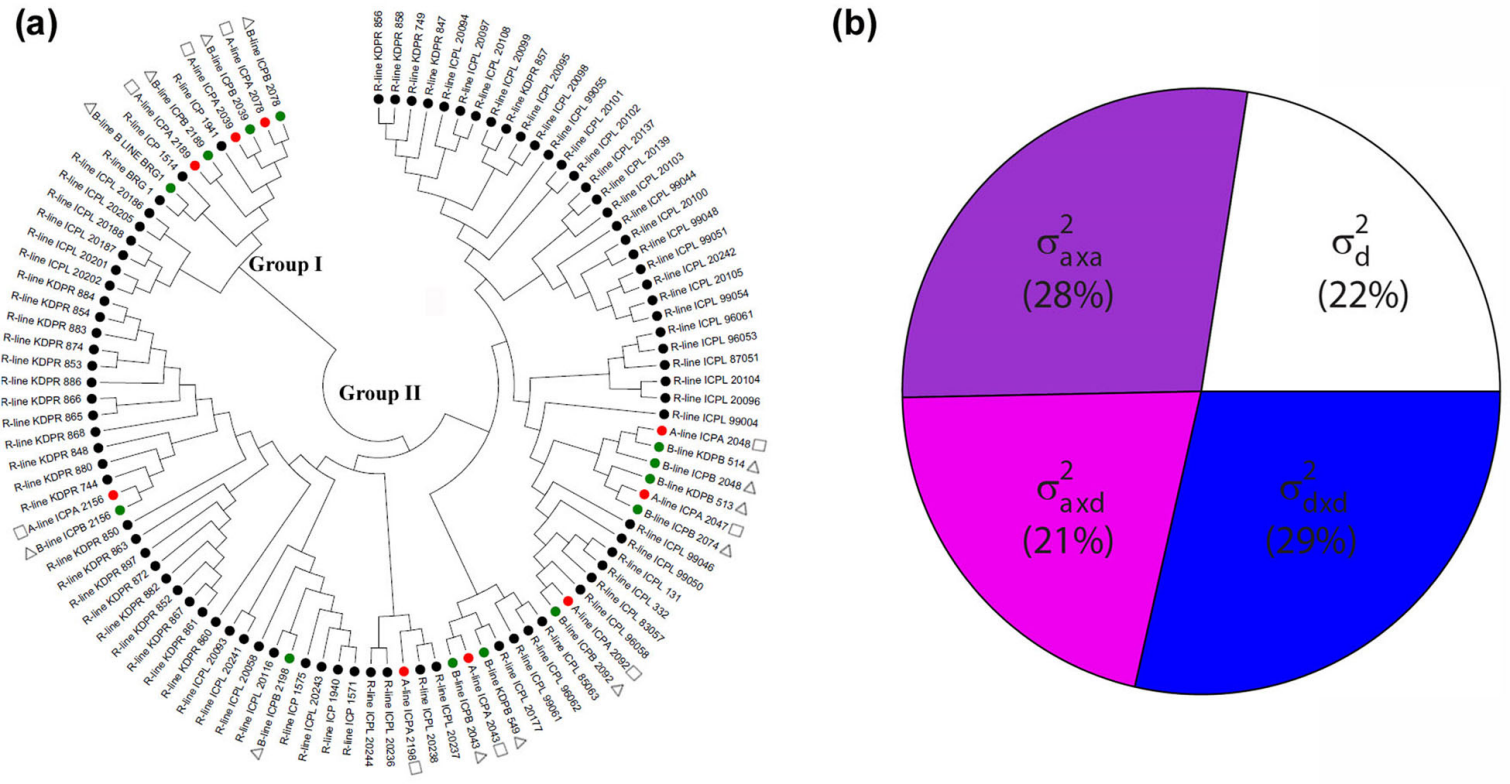
An overview of the genetic relationships in the parental lines and the relative contribution of the genetic components to mid‐parent heterosis for grain yield. (a) Genetic relationship analysis of 104 parental lines of the hybrids: nine cytoplasmic male sterile (CMS; A‐lines, in red and marked with square boxes), 13 maintainers (B‐lines, in green and marked with triangles) and 82 restorer (R‐lines, in black colour). The analysis used single nucleotide polymorphisms (SNPs) detected in the whole genome resequencing data. (b) Relative contributions of the genetic components of mid‐parent heterosis for grain yield estimated via Bayes generalized linear regression

The sequence variation data were used to understand the genetic relationships among the parental lines. Analyses based on pairwise dissimilarities via neighbor joining revealed two distinct groups (Figure 1a). Group I contained three A‐lines, four B‐lines, and nine R‐lines. Group II included six A‐lines, nine B‐lines, and the remaining 73 R‐lines. Many of the A‐lines were assigned as the closest neighbor to their respective B‐lines, supporting the notion of iso‐nuclear lines. The extent of similarity between A‐ and B‐ lines also corresponded to backcross generations (i.e., A‐lines with higher number of backcrosses showed higher similarity to their corresponding B‐lines). Pairwise genome‐wide fixation index (F_ST_) values (Weir & Cockerham, 1984) also revealed the close relationship between A‐lines and B‐lines (F_ST_ = 0.09). In contrast, pronounced differentiation was observed between the A‐lines and R‐lines (F_ST_ = 0.26), and between B‐lines and R‐lines (F_ST_ = 0.29) (Supplemental Table S7). The diversity among the parents makes this panel suitable for studying the genetic basis and exploitation of heterosis.

### 3.3 Dissecting the genetic architecture of heterosis via WGRS data

As the first step, we investigated the relationship between the genetic diversity and heterosis. The correlation between the Rogers’ distance in parental lines and the MPH was very low and nonsignificant (*r* = 0.05, with a *P*‐value of .35). Even if we restricted the analysis to the hybrids with a MPH above 50 g per plant, the correlation was still weak (*r* = 0.26, with a *P*‐value of .02).

Therefore, a genome‐wide prediction model including dominance and digenic epistatic effects (Jiang et al., 2017) was applied to partition the genetic variance components for MPH. The differences between the proportions of genetic variance explained by the four types of genetic effect were not large (Figure 1b). Additive‐by‐additive and dominance‐by‐dominance epistasis explained a slightly larger proportion of genetic variance than dominance and additive‐by‐dominance epistasis did. However, the relative contributions to MPH of the different genetic effects represent only a rough estimate because of the confounding effects of the different genetic effects, as indicated by the high correlations between the marker‐derived kinship matrices for dominance and digenic epistasis (Supplemental Table S8). The confounding effect was further substantiated by the prediction abilities of MPH evaluated in a fivefold cross‐validation (Table 2). The prediction ability was highest for the model including dominance and additive‐by‐additive effects. Nevertheless, with additive‐by‐additive effects alone in the model, a similar prediction ability could be reached. Adding additive‐by‐dominance or dominance‐by‐dominance effects into the model could not further increase the prediction ability.

**TABLE 2:** Cross‐validated prediction abilities for mid‐parent heterosis (MPH) obtained by a genome‐wide prediction model including different genetic effects

**Model** ^a^	**Prediction ability** ^b^
D	0.222 (0.030)
AA	0.238 (0.022)
D + AA	0.240 (0.027)
D + AA + AD	0.239 (0.027)
D + AA + AD + DD	0.236 (0.026)

^a^ D, dominance; AA, additive‐by‐additive; D + AA, dominance + additive‐by‐additive; D + AA + AD, dominance + additive‐by‐additive + additive‐by‐dominance; D + AA + AD + DD, dominance + additive‐by‐additive + additive‐by‐dominance + dominance‐by‐dominance; ^b^ The numbers in parenthesis indicate the SD estimated in 20 replicates of fivefold cross‐validation.

**FIGURE 2:**
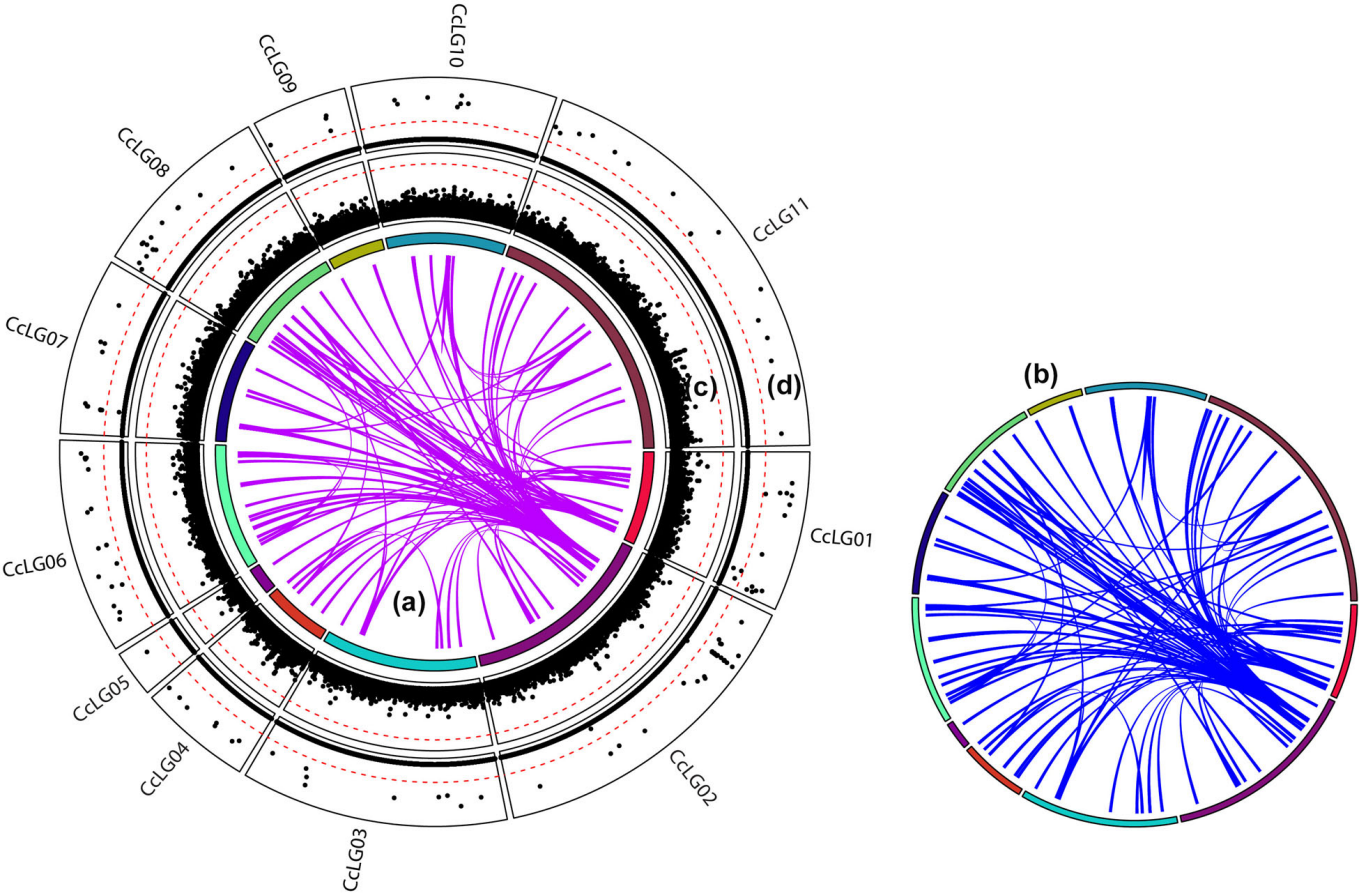
The genetic architecture of mid‐parent heterosis for grain yield in pigeonpea. Pigeonpea chromosomes or pseudomolecules are indicated as bars on the inner circle. Colored links in the center of the circles represent significant digenic epistatic interactions: (a) additive‐by‐additive and (b) additive‐by‐dominance. (c) Manhattan plot for the dominance effects identified in the genome‐wide association study (GWAS). (d) Manhattan plot for the heterotic effects identified in the GWAS. Significant thresholds (*P* < .05 after Bonferroni–Holm correction for multiple testing) are indicated as red dashed lines

To elucidate the genetic architecture of grain yield heterosis in further detail, the quantitative genetic framework (Jiang et al., 2017) was implemented. In the first step, GWAS was performed to detect significant dominance and digenic epistatic effects among all 128,067 SNPs. We observed no significant dominance effect even with a liberal threshold of false discovery rate <0.1 despite the dominance effects explaining 22% of the genetic variance. We also performed a GWAS for the dominance effects directly, based on the hybrid performance (Supplemental Figure S3). With the same liberal threshold, two significant marker loci were identified. Nevertheless, their dominance effects together only explained 4.1% of the phenotypic variance of MPH. Thus, it was likely that many loci with small dominance effects contributed to MPH. With a very stringent threshold of *P* < .05 after Bonferroni correction for multiple testing, 192 additive‐by‐additive and 188 dominance‐by‐dominance effects were significant, although no additive‐by‐dominance effects passed the threshold (Figure 2, Supplemental Table S9, Supplemental Table S10). The SNP pairs with significant epistatic effects were not independent of each other because of linkage disequilibrium. By applying principal component analysis, we found that the number of independent SNP pairs was 36 for additive‐by‐additive effects and 50 for dominance‐by‐dominance effects. In the second step, the significant effects detected in the GWAS were integrated to heterotic effects for each SNP, defined as a complex combination of the dominance effect of the SNP itself and the epistatic interaction effects with the entire genetic background (see the Materials and Methods for details). As the final step, the significance of the heterotic effect for each SNP was tested by a permutation test for the Pearson correlation coefficient between the observed MPH values and those predicted by the heterotic effect of the SNP alone. We observed 129 SNPs that showed significant heterotic effects (Figure 2, Supplemental Table S11), whereas the number of independent SNPs was estimated to be 40 (Supplemental Table S12). Together, these SNPs explained 27% of the phenotypic variance of MPH, indicating that each SNP had a rather small effect. Out of the 40 SNPs with significant heterotic effects, only five were present in genic regions that played a role in molecular functions; the remaining 35 were found in intergenic regions (Supplemental Table S12).

### 3.4 Investigating the contribution of CNVs to heterosis

Complementation of allelic variation could be a contributor to heterosis, especially in crosses between fixed parental lines with more CNVs. To study the contributions of CNVs to heterosis, 869 CNVs identified across the parental lines of the hybrids were considered via a multiallelic model (Jiang et al., 2017). In particular, the CNV at each site was considered as a multiallelic marker, and the dominance effects (i.e., the interaction effects between different alleles) within each CNV was tested (for details, see the Materials and Methods). Therefore, we did not assume linear effects with respect to the number of copies. As a result, we identified 52 CNVs that significantly contributed to MPH at *P* < .05 after Benjamini–Hochberg correction (false discovery rate) (Supplemental Table S13). However, these CNVs together explained only 0.4% of the phenotypic variance in MPH.

The results of the previous two subsections indicated that the genetic architecture of heterosis for grain yield in pigeonpea is complex. MPH is caused by many loci with small dominance and digenic epistatic effects, with very small effects resulting from CNVs. This conclusion made it difficult to apply the knowledge of genetic architecture of heterosis directly to improve hybrid breeding. Thus, we focused on hybrid performance and used genome‐wide predictions to identify a genetically complementary high‐yielding heterotic pattern.

### 3.5 Establishing a high‐yielding heterotic pattern

Since the number of individuals in the training population is an important factor affecting the accuracy of genome‐wide prediction, we combined the dataset analyzed so far with another dataset consisting of 292 inbred lines of pigeonpea generated in a previous study (Varshney et al., 2017), which had also been evaluated for grain yield in multienvironment trials and sequenced at the whole‐genome level. Thus, in total, there were 396 inbred lines (292 + 104 hybrid parental lines) and 435 hybrids. Combining the WGRS data of the two panels resulted in 8,554,715 SNPs. After quality control for missing values (<5%) and minor allele frequency (>2.5%), 725,701 high‐quality markers were used for further analyses (Supplemental Figure S4). The average Rogers’ distance among the 396 lines was 0.22, with a range from 0.04 to 0.44 (Supplemental Figure S5).

To investigate whether the performance of grain yield in hybrids can be reliably predicted by WGRS data, we applied the ridge regression best linear unbiased prediction model, including the additive and dominance genotypic values (Zhao et al., 2015), and considered different cross‐validation scenarios. More precisely, the training population comprised 7 female and 35 male parental lines and 160 of their hybrid progeny as well as 292 additional inbred lines. The remaining hybrids were divided into three test populations with different degrees of relatedness to the training population (see the Materials and Methods for details). We observed that for the T2 scenario, in which both parents of the hybrid being predicted were included in the training population, the prediction accuracy for grain yield was 0.24 (Supplemental Figure S6), which is lower than was previously reported for maize (Technow et al., 2014) and wheat (Jiang et al., 2017) but higher than for rice (Xu et al., 2014).

As the next step, we used all 396 lines and 435 hybrids as the training population and predicted the yield performance of all 78,210 possible single‐cross hybrids derived from the 396 lines. Since all parental lines were included in the training population, similar prediction accuracy to the cross‐validated T2 scenario was expected. According to the predicted values, the average yield of the 0.1% top‐yielding hybrids was 119.6 g per plant, which was 65% higher than the average yield of all hybrids, and 191% higher than the average yield of all inbred lines. Interestingly, only 12 of the 78 hybrids have been tested so far. The remaining outstanding hybrids, whose parental lines have not yet been used for hybrid breeding, are therefore interesting targets for further intensive field evaluations (Supplemental Figure S7). We then identified a group of 39 lines whose hybrid progeny exhibited high yield (Supplemental Table S14) by applying hierarchical clustering to the predicted yield performance of all 78,210 hybrids (Supplemental Figure S8A). The average yield of hybrids from this group was 85.8 g per plant, which was 19% higher than the average yield of all tested hybrids.

Identifying heterotic groups, defined as groups of genetically distinct genotypes which display high hybrid performance when crossed with each other, is key to accelerating hybrid breeding. On the basis of the predicted performance of all 78,210 hybrids, we implemented a simulated annealing algorithm (Zhao et al., 2015) to generate two potential high‐yield heterotic groups, each consisting of 20 lines (Supplemental Table S14). The average yield of the hybrids derived by crossing parental lines in the two groups was 90.4 g per plant, which was 25% higher than average yield of all tested hybrids (Supplemental Figure S8B). Twenty‐nine of the 40 lines were also selected by the clustering algorithm, but the performance of heterotic groups identified by the simulated annealing algorithm was, on average, 5% higher than the single group selected by the clustering algorithm. We expect that the high‐yielding heterotic groups detected in our study could serve as a cornerstone for a further in‐depth search for the most promising heterotic groups for pigeonpea.

## 4 DISCUSSION

Breeding hybrids in various crop species has proven to be one of the most efficient ways to increase grain yield (Schnable & Springer, 2013), though there are differences in terms of the extent of realized heterosis depending on the crop species and also differences within the crop species. Therefore, in conventional breeding, a large number of hybrid crosses have to be screened to select the best parental combination maximizing yield performance. However, many parental combinations can be disregarded because of relatedness between the parents or the contributions of different dominance, overdominance and epistasis interactions in heterosis. Instead, we can rely on combining ability or performance per se. An integrated approach was required that can explain the molecular basis of heterosis and select the best crossing combinations. Therefore, in the present study, we used WGRS data to understand the molecular basis of heterosis and define heterotic patterns through genome‐wide predictions of the hybrid performance. Our results indicated that the genetic architecture of heterosis for grain yield in pigeonpea is complex and is caused by many loci with small dominance and digenic epistatic effects with very small effects resulting from CNVs. Although some previous studies dissecting the genetic basis of heterosis in maize and rice favored incomplete dominance instead of epistasis (Gerke et al., 2015; Huang et al., 2016), it has also been reported in many studies that heterosis is caused by an accumulation of dominance and epistatic effects (Hua et al., 2003; Radoev et al., 2008) or even mainly by epistasis (Melchinger et al., 2007; Yu et al., 1997). In addition, it was hypothesized that the genetic basis of heterosis was mainly contributed by dominance in open‐pollinated species, whereas for heterosis in self‐pollinating species, the additive‐by‐additive epistasis played a major role (Garcia et al., 2008). This was supported by a recent study on grain yield heterosis in wheat (Jiang et al, 2017). Thus, our results are not just specific to pigeonpea but also apply to other crops that are often cross‐pollinated. The WGRS data also provided us with an opportunity to roll out “apparent epistasis” (Wood et al., 2014). Since additive effects do not contribute to MPH by definition, the only possible way that apparent epistasis occurs is that an “epistatic pseudo‐marker” (Lachowiec et al., 2015) is highly correlated with a marker encoding a dominance effect. However, as WGRS data were used for GWAS and we observed no significant dominance effect, it seems that apparent epistasis did not occur in our study.

Another milestone achieved in the present study was the deployment of WGRS data for predicting yield in hybrids. The prediction accuracy of genome‐wide prediction, especially for complex traits, has been found to be high with a large number of markers from WGRS rather than a few thousands of markers (Li et al., 2018; Meuwissen & Goddard, 2010; Ober et al., 2012). It can be expected that the divergent heterotic groups identified in the present study will promote, in the long term, the role of additive versus dominance genetic variance (Reif et al., 2007), which will ultimately boost reciprocal recurrent selection efficiency in pigeonpea. Furthermore, additive effects have been found to be reliable for increasing the accuracy of genome‐wide prediction in maize (Technow et al., 2014). By applying genome‐wide prediction and a simulated annealing algorithm in the present study, we have identified an interesting group of 29 lines exhibiting high hybrid performance when crossed with each other. In view of the 396 lines used in the present study, the identified set of 29 lines looks quite small. However, any hybrid breeding program should focus on selecting parental lines that will maximize the response for selection. Interestingly, the effective number of parental lines in hybrid wheat breeding was 16 per heterotic group (Zhao et al., 2015); similarly it was around 16 in maize (van Heerwaarden et al., 2012). Therefore, the identified set of 29 lines in pigeonpea will provide a strong platform for sustainable long‐term selection gain in a hybrid breeding program. This study was based on elite lines of pigeonpea and thus the results are immediately relevant for breeding hybrid pigeonpea. This study also provides a better understanding of heterosis in a complex yield trait, which may allow new schemes for handling heterosis in a precise manner. We believe this study will also serve as a model for other crops where hybrid breeding efforts are in progress regarding the use of WGRS data in quantitative genetic framework for facilitating hybrid breeding and achieving the quantum jump in crop yield for ensuring global food security.

## CONFLICT OF INTEREST

The authors declare that they have no competing interests.

## AUTHOR CONTRIBUTIONS

Rachit K. Saxena: conceptualization, formal analysis, investigation, project administration, resources, supervision, writing—original draft, writing—review and editing. Yong Jiang: conceptualization, formal analysis, writing—review and editing. Aamir W Khan: data curation, formal analysis. Yusheng Zhao: formal analysis, writing—review and editing. Vikas Kumar Singh: data curation, writing—review and editing. Abhishek Bohra: formal analysis, writing—review and editing. Muniswamy Sonappa: data curation, writing—review and editing. Abhishek Rathore: data curation. C. V. Sameer Kumar: data curation, writing—review and editing. Kulbhushan Saxena: methodology, writing—review and editing. Jochen Reif: conceptualization, data curation, formal analysis, software, supervision, writing—review and editing. Rajeev K. Varshney: conceptualization, formal analysis, funding acquisition, investigation, project administration, resources, supervision, writing—original draft.

## Supporting information

Supplemental Figure S1. Distribution of the BLUEs across environments for nine traits for hybrid and their parent lines.
Supplemental Figure S2. Distribution of mid‐parent heterosis values for grain yield
Supplemental Figure S3. Manhattan plots of the GWAS for dominance effects based on (A) MPH and (B) hybrid performance. The red horizontal lines indicate the threshold of P < .05 after Bonferroni correction. The red dashed line in Panel B indicated the false discovery rate threshold of <0.1
Supplemental Figure S4. Minor allele frequency and heterozygosity rate for all 725,701 markers
Supplemental Figure S5. Heat map for 1 minus the Roger's distance matrix (1 – RD) of all 396 lines
Supplemental Figure S6. Prediction accuracy for hybrids obtained from the hybrid and inbred lines for various validation sets exploiting additive and dominance effects for all nine traits. T0, no common parent with the estimation set; T1, one parent in common with the estimation set; T2, two parents in common with the estimation set
Supplemental Figure S7. Venn plot of the parental lines of the 78 most outstanding predicted hybrids and all tested hybrids
Supplemental Figure S8. Heat plot of predicted hybrid performance ordered via complete linkage clustering (A) and ordered by the developed simulated annealing algorithm (B)

**Supplemental Table S1**. Raw sequencing data generated and the alignment statistics of 104 hybrid parental lines

Supplemental Table S2. Estimates of the variance components and heritability for grain yield per plant (g per plant), days to 50% flowering (DF), days to maturity 75% (DM), plant height (PH, in cm), number of pods per plant, 100‐seed weight (Seedwt100, in g), number of seeds per pod, number of primary branches per plant, and number of secondary branches per plant of hybrid data. Here, $\sigma_{\mathrm{GC}A_{f}}^{2}$ is the variance component of the general combining ability (GCA) of female parental lines, $\sigma_{\mathrm{GC}A_{m}}^{2}$ is the variance component of the GCA for the male parental lines, and $\sigma_{\mathrm{SCA}}^{2}$ is the variance components of the specific combining ability (SCA) of the hybrids

Supplemental Table S3. Copy number variations (≥1 kb) identified in the hybrid parental lines

Supplemental Table S4. Genome‐wide variation among nine CMS lines
Supplemental Table S5. Genome‐wide variation among 13 maintainer lines
Supplemental Table S6. Genome‐wide variation among 82 restorer lines

Supplemental Table S7. Fixation index (FST) values estimated in pairwise combinations

**Supplemental Table S8**. Correlations between the marker‐derived kinship matrices for dominance and digenic epistatic effects

Supplemental Table S9. Markers with significant additive‐by‐additive effects detected

**Supplemental Table S10**. Markers with significant dominance‐by‐dominance effects detected

**Supplemental Table S11**. Markers with significant heterotic QTL detected in pigeonpea

**Supplemental Table S12**. Details of 40 independent SNPs showing heterotic effects.

**Supplemental Table S13**. Details of 52 CNVs showing heterotic effects

**Supplemental Table S14**. Heterotic groups detected by hierarchical clustering and the simulated annealing algorithm method

## Data Availability

The WGRS dataset generated and analyzed in the current study is available from NCBI under BioProject accession number PRJNA575817.
