# Supplementary material for: Characterization of heterosis and genomic prediction‐based establishment of heterotic patterns for developing better hybrids in pigeonpea
Source: Plant Genome. 2021 Aug 1;14(3):e20125. doi: 10.1002/tpg2.20125 (PMC12807029; doi:10.1002/tpg2.20125)
Supplement: Supplementary file 1 [file TPG2-14-e20125-s006.docx]

**Supplemental Figure 1.** Distribution of the BLUEs across environments for 9 traits for hybrid and their parent lines

**Supplemental Figure 2**. Distribution of midparent heterosis values for grain yield.

**Supplemental Figure 3.** The Manhattan plots of the GWAS for dominance effects based on A) the mid-parent heterosis and B) the hybrid performance. The red horizontal lines indicated the threshold of P < 0.05 after Bonferroni correction. The red dashed line in panel B indicated the threshold of FDR < 0.1.

**Supplemental Figure 4.** Minor allele frequency and heterozygosity rate for all 725,701 markers

**Supplemental Figure 5.** Heat map for one minus the Roger’s distance matrix (1-RD) of all 396 lines

**Supplemental Figure 6.** Prediction accuracy for hybrids obtained using both hybrid and inbred lines for various validation sets (T0: no common parent with estimation set, T1: one common parent with estimation set, and T2: two common parents with estimation set) exploiting additive and dominance effects for all nine traits.

**Supplemental Figure 7.** Venn plot of parental lines of the 78 most outstanding predicted and all tested hybrids.

(A)

(B)

**Supplemental Figure 8.** Heat plot of predicted hybrid performance ordered using complete linkage clustering (A) and ordered based on the developed simulated annealing algorithm (B).

**Supplemental Table 1.** Raw sequencing data generated and alignment statistics of 104 hybrid parental lines.

Separate excel file has been provided.

**Supplemental Table 2.** Estimates of variance components and heritability for grain yield per plant (gram per plant), days to 50% flowering (DF; days), days to maturity 75% (DM; days), plant height (PH; cm), number of pods per plant, 100 seed weight (Seedwt100; g), number of seeds per pod, number of primary branches per plant, and number of secondary branches per plant of hybrid data. $\sigma_{\mathrm{GCA}_{f}}^{2}$ is the variance component of the general combining ability (GCA) of female parental lines, $\sigma_{\mathrm{GCA}_{m}}^{2}$ is the variance component of the GCA for the male parental lines, $\sigma_{\mathrm{SCA}}^{2}$ is the variance components of the specific combining ability (SCA) of the hybrids.

| Variance components | Heritability | | $\sigma_{\mathrm{GCA}_{f}}^{2}$ | $\sigma_{\mathrm{GCA}_{m}}^{2}$ | $\sigma_{\mathrm{SCA}}^{2}$ | $\sigma_{E}^{2}$1 |
| --- | --- | --- | --- | --- | --- | --- |
| Days to 50% flowering | 0.82 | ${31.36}^{***}$ | | ${1.61}^{***}$ | ${7.132}^{***}$ | 35.24 |
| Days to maturity 75% | 0.78 | ${34.54}^{***}$ | | ${1.87}^{***}$ | ${6.24}^{***}$ | 47.64 |
| Plant height | 0.37 | ${7.56}^{***}$ | | ${7.54}^{**}$ | ${13.03}^{**}$ | 195.62 |
| Pods/plant | 0.87 | ${2487.30}^{***}$ | | ${1682.69}^{**}$ | ${16953.87}^{***}$ | 12514.02 |
| 100 seed weight | 0.87 | ${0.35}^{***}$ | | $0.05$ | ${1.11}^{***}$ | 0.878 |
| Seeds/pod | 0.20 | 0.004 | | 0.003 | 0 | 0.12 |
| Grain yield/plant | 0.90 | ${94.11}^{***}$ | | ${70.88}^{***}$ | ${816.16}^{***}$ | 454.70 |
| Primary branches/plant | 0.55 | ${1.17}^{***}$ | | 0.098 | ${1.79}^{***}$ | 10.11 |
| Secondary branches/plant | 0.75 | ${6.40}^{***}$ | | 0.449 | ${26.82}^{***}$ | 45.50 |

1 Variance of the residual, $***,$p<0.01, $**$ p<0.05,$*$ p<0.1 .

**Supplemental Table 3.** Copy number variations (≥1kb) identified in hybrid parental lines.

Separate excel file has been provided.

**Supplemental Table 4.** Genome wide variation among nine CMS lines.

| Linkage group | SNPs | Indels | Total variant |
| --- | --- | --- | --- |
| CcLG01 | 47,531 | 9,030 | 56,561 |
| CcLG02 | 76,377 | 14,906 | 91,283 |
| CcLG03 | 50,273 | 10,486 | 60,759 |
| CcLG04 | 29,784 | 6,218 | 36,002 |
| CcLG05 | 11,194 | 2,191 | 13,385 |
| CcLG06 | 46,451 | 9,489 | 55,940 |
| CcLG07 | 40,521 | 8,075 | 48,596 |
| CcLG08 | 44,086 | 8,510 | 52,596 |
| CcLG09 | 24,627 | 4,497 | 29,124 |
| CcLG10 | 50,125 | 8,439 | 58,564 |
| CcLG11 | 107,888 | 19,231 | 127,119 |
| Total | 528,857 | 101,072 | 629,929 |

**Supplemental Table 5.** Genome wide variation among 13 maintainer lines.

| Linkage group | SNPs | Indels | Total variant |
| --- | --- | --- | --- |
| CcLG01 | 53,474 | 10,077 | 63,551 |
| CcLG02 | 85,057 | 16,479 | 101,536 |
| CcLG03 | 59,427 | 12,527 | 71,954 |
| CcLG04 | 32,496 | 6,804 | 39,300 |
| CcLG05 | 12,776 | 2,503 | 15,279 |
| CcLG06 | 54,193 | 10,989 | 65,182 |
| CcLG07 | 44,796 | 8,840 | 53,636 |
| CcLG08 | 50,115 | 9,678 | 59,793 |
| CcLG09 | 29,306 | 5,304 | 34,610 |
| CcLG10 | 56,396 | 9,418 | 65,814 |
| CcLG11 | 119,853 | 21,252 | 141,105 |
| Total | 597,889 | 113,871 | 711,760 |

**Supplemental Table 6.** Genome wide variation among 82 restorer lines.

| Linkage group | SNPs | Indels | Total variant |
| --- | --- | --- | --- |
| CcLG01 | 108,569 | 20,588 | 129,157 |
| CcLG02 | 156,641 | 30,354 | 186,995 |
| CcLG03 | 107,840 | 23,012 | 130,852 |
| CcLG04 | 61,646 | 12,640 | 74,286 |
| CcLG05 | 21,791 | 4,324 | 26,115 |
| CcLG06 | 101,894 | 21,522 | 123,416 |
| CcLG07 | 90,728 | 17,869 | 108,597 |
| CcLG08 | 88,901 | 17,588 | 106,489 |
| CcLG09 | 59,106 | 10,479 | 69,585 |
| CcLG10 | 111,717 | 18,940 | 130,657 |
| CcLG11 | 222,236 | 39,850 | 262,086 |
| Total | 1,131,069 | 217,166 | 1,348,235 |

**Supplemental Table 7.** F_ST_ values estimated in pair-wise combinations.

Separate excel file has been provided.

**Supplemental Table 8.** Correlations between the marker-derived kinship matrices for dominance and digenic epistatic effects

|  | D | AA | AD | DD |
| --- | --- | --- | --- | --- |
| D |  | 0.93 | 0.95 | 0.98 |
| AA |  |  | 0.97 | 0.97 |
| AD |  |  |  | 0.95 |
| DD |  |  |  |  |

**Supplemental Table 9.** Markers with significant additive x additive effects detected.

Separate excel file has been provided.

**Supplemental Table 10.** Markers with significant dominance x dominance effects detected.

Separate excel file has been provided.

**Supplemental Table 11.** Markers with significant heterotic QTL in pigeonpea detected.

Separate excel file has been provided.

**Supplemental Table 12.** Details on 40 independent SNPs showing heterotic effects.

Separate excel file has been provided.

**Supplemental Table 13.** Supplemental Table 13. Details on 52 CNVs showing heterotic effects.

Separate excel file has been provided.

**Supplemental Table 14.** Heterotic groups detected by hierarchical clustering and simulated annealing algorithm method

| Hierarchical clustering | Simulated annealing algorithm | |
| --- | --- | --- |
|  | ***Group1*** | ***Group2*** |
| ICP1071 | ICP1575 | ICP1071 |
| ICP11015 | ICP3451 | ICP11015 |
| ICP11406 | ICP8863 | ICP11406 |
| ICP1156 | ICPA2043 | ICP12515 |
| ICP13662 | ICPA2092 | ICP14524 |
| ICP14770 | ICPB2043 | ICP14770 |
| ICP1575 | ICPL20058 | ICP4029 |
| ICP1940 | ICPL20095 | ICP6971 |
| ICP2746 | ICPL20096 | ICP7507 |
| ICP3451 | ICPL20098 | ICP7532 |
| ICP3576 | ICPL20101 | ICP8152 |
| ICP4029 | ICPL20102 | ICP8211 |
| ICP6123 | ICPL20105 | ICPA2047 |
| ICP6971 | ICPL20237 | ICPA2048 |
| ICP7532 | ICPL83057 | ICPA2189 |
| ICP8211 | ICPL99048 | ICPB2047 |
| ICP8255 | ICPL99050 | ICPB2048 |
| ICP9499 | ICPL99051 | ICPL20188 |
| ICP9671 | ICPL99054 | KDPB513 |
| ICP995 | ICPL99055 | KDPB514 |
| ICPA2043 |  |  |
| ICPA2047 |  |  |
| ICPA2048 |  |  |
| ICPA2189 |  |  |
| ICPB2043 |  |  |
| ICPB2047 |  |  |
| ICPB2048 |  |  |
| ICPL20095 |  |  |
| ICPL20096 |  |  |
| ICPL20098 |  |  |
| ICPL20101 |  |  |
| ICPL20105 |  |  |
| ICPL20188 |  |  |
| ICPL83057 |  |  |
| ICPL99050 |  |  |
| ICPL99051 |  |  |
| ICPL99054 |  |  |
| KDPB513 |  |  |
| KDPB514 |  |  |

**Supplemental Note**

**A. An insight to the grain yield of hybrid pigeonpea through historical data**

Available historical yield data on 44 hybrid parental lines (9 B-lines and 35 R-lines) and thereof derived 68 hybrids from 2 to 16 environments during past nine years (2004-2012) (Supplemental note A Table 1) were analyzed. The yield of B-lines was ranged from 623.71 Kg/ha to 2046.29 Kg/ha and yield of R-lines ranged from 179.62 Kg/ha to 2448.46 Kg/ha. Analysis of hybrid parental lines revealed that average yield of R-lines with 1709.64 Kg/ha were comparatively higher in comparison to the average yield of B-lines with 1302.31 Kg/ha (Supplemental note A Table 1). Mid parent heterosis (MPH) for the hybrids was ranged from -50.73 to 183.67 % (Supplemental note A Table 2).

**Supplemental note A Table 1.** Parents and their key agronomic performances across environments

| Parent | Environments | Days to 50% flowering (days) | Plant height (cm) | 100 seed weight (g) | Yiled (Kg/ha) |
| --- | --- | --- | --- | --- | --- |
| ICPB 2039 | 2 | 80.00^a^ | 95.00^a^ | 9.00^b-h^ | 1468.51^b-i^ |
| ICPB 2043 | 6 | 114.17^b-e^ | 173.33^b-d^ | 10.45^h-o^ | 1580.60^c-j^ |
| ICPB 2047 | 8 | 123.00^c-j^ | 225.00^d-f^ | 10.88^j-p^ | 1672.53^c-j^ |
| ICPB 2048 | 4 | 124.50^d-j^ | 235.00^ef^ | 11.97^o-r^ | 2046.29^h-j^ |
| ICPB 2078 | 6 | 114^b-d^ | 128.33^ab^ | 13.51^s^ | 1094.23^b-h^ |
| ICPB 2092 | 6 | 120.00^b-h^ | 223.33^d-f^ | 9.70^d-k^ | 1502.70^b-h^ |
| ICPB 2156 | 2 | 72.50^a^ | 137.50^a-c^ | 8.35^b-d^ | 824.07^a-e^ |
| ICPB 2189 | 6 | 114.67^b-f^ | 213.33^d-f^ | 9.98^e-l^ | 908.18^a-f^ |
| ICPB 2198 | 4 | 127.40^h-m^ | 218.75^d-f^ | 7.97^b^ | 623.71^ab^ |
| ICPL 20093 | 10 | 128.10^g-l^ | 221.00^d-f^ | 11.43^l-r^ | 1648.17^d-j^ |
| ICPL 20058 | 12 | 119.75^b-h^ | 202.58^d-f^ | 9.25^b-i^ | 1940.73^g-j^ |
| ICPL 20094 | 10 | 134.80^j-o^ | 228.50^d-f^ | 10.28^h-m^ | 1827.18^f-h^ |
| ICPL 20095 | 10 | 122.30^c-i^ | 203.50^d-f^ | 10.07^f-m^ | 1702.35^e-j^ |
| ICPL 20096 | 10 | 127.80^g-l^ | 214.00^d-f^ | 11.63^m-r^ | 1702.29^e-j^ |
| ICPL 20097 | 2 | 138.00^l-o^ | 230.00^ef^ | 11.05^j-p^ | 1978.70^h-j^ |
| ICPL 20098 | 10 | 127.40^h-n^ | 216.50^d-f^ | 12.90^rs^ | 1806.26^f-j^ |
| ICPL 20099 | 10 | 126.60^f-l^ | 225.00^d-f^ | 11.61^m-r^ | 1770.80^f-j^ |
| ICPL 20100 | 2 | 140.00^m-o^ | 225.00^d-f^ | 9.70^d-k^ | 2016.66^h-j^ |
| ICPL 20101 | 2 | 138.00^l-o^ | 235.00^ef^ | 11.65^m-r^ | 974.99^a-g^ |
| ICPL 20102 | 10 | 126.80^f-l^ | 223.00^d-f^ | 11.00^j-p^ | 1694.46^e-j^ |
| ICPL 20103 | 2 | 141.00^m-o^ | 235.00^ef^ | 12.35^p-s^ | 1438.87^b-i^ |
| ICPL 20104 | 10 | 127.40^g-l^ | 219.00^d-f^ | 12.64^q-s^ | 1812.87^f-j^ |
| ICPL 20105 | 2 | 145.00^o^ | 245.00^f^ | 12.95^q-s^ | 1411.10^b-i^ |
| ICPL 20108 | 8 | 126.38^e-l^ | 224.38^d-f^ | 10.05^e-m^ | 1978.70^h-j^ |
| ICPL 20116 | 10 | 125.70^d-k^ | 199.50^d-f^ | 10.20^g-m^ | 1821.58^f-j^ |
| ICPL 20137 | 6 | 142.00^no^ | 226.67^d-f^ | 11.63^m-r^ | 622.42^ab^ |
| ICPL 20177 | 4 | 125.50^d-k^ | 230.00^ef^ | 11.37^i-r^ | 1862.00^g-j^ |
| ICPL 20186 | 2 | 133.75^k-o^ | 223.75^d-f^ | 8.57^b-f^ | 1383.23^b-i^ |
| ICPL 20201 | 2 | 143.00^no^ | 245.00^f^ | 9.70^d-k^ | 1462.03^b-i^ |
| ICPL 20202 | 4 | 126.50^e-l^ | 220.00^d-f^ | 10.87^j-p^ | 2005.55^h-j^ |
| ICPL 20205 | 4 | 125.50^d-k^ | 195.00^d-f^ | 8.35^b-d^ | 2138.88^ij^ |
| ICPL 20243 | 2 | 117.00^b-g^ | 292.50^g^ | 5.7^a^ | 179.62^a^ |
| ICPL 332 | 6 | 117.50^b-g^ | 197.50^d-f^ | 8.50^b-e^ | 2448.46^j^ |
| ICPL 96053 | 6 | 123.17^c-j^ | 183.33^c-e^ | 12.40^p-s^ | 1892.28^g-j^ |
| ICPL 96058 | 6 | 119.83^b-h^ | 200.00^d-f^ | 10.15^f-m^ | 1892.28^g-j^ |
| ICPL 96061 | 8 | 122.21^c-i^ | 210.00^d-f^ | 10.60^i-o^ | 1672.37^c-j^ |
| ICPL 99004 | 8 | 109.62^b^ | 194.37^d-f^ | 8.08^bc^ | 677.36^a-c^ |
| ICPL 99044 | 2 | 122.00^c-i^ | 207.50^d-f^ | 12.20^p-s^ | 1973.14^h-j^ |
| ICPL 99046 | 4 | 127.40^h-m^ | 222.50^d-f^ | 11.20^k-q^ | 1512.90^b-j^ |
| ICPL 99048 | 2 | 122.00^c-i^ | 225.50^def^ | 8.70^b-g^ | 2027.77^h-j^ |
| ICPL 99051 | 2 | 120.00^b-h^ | 200.00^d-f^ | 11.45^l-r^ | 2199.99^h-j^ |
| ICPL 99054 | 2 | 120.50^b-h^ | 237.50^ef^ | 11.90^n-r^ | 2212.03^h-j^ |
| ICPL 99055 | 2 | 127.00^f-l^ | 220.00^d-f^ | 12.70^q-s^ | 2439.81^j^ |
| ICPL 99061 | 2 | 120.00^b-h^ | 215.00 | 9.40^d-g^ | 1925.92^g-h^ |

Mean data over environment

Differenet letters means signifcant difference at 0.05 lelvel by Duncan’s multiple range test

**Supplemental note A Table 2.** Average yield performance 68 hybrid (single location) in comparison to the respective parents

| Hybrid | Env | Average yield (Kg/ha) | Parent 1 | Average yield (Kg/ha) | Parent 2 | Average yield (Kg/ha) | Mid parent yield (Kg/ha) | Relative mid-parent heterosis (%) |
| --- | --- | --- | --- | --- | --- | --- | --- | --- |
| ICPH 2672 | 2 | 5075 | ICPA 2043 | 1581 | ICPL 20202 | 2006 | 1793 | 183.00 |
| ICPH 2673 | 14 | 2013 | ICPA 2043 | 1581 | ICPL 20205 | 2139 | 1860 | 8.24 |
| ICPH 2675 | 4 | 3896 | ICPA 2043 | 1581 | ICPL 20201 | 1462 | 1521 | 156.13 |
| ICPH 2680 | 8 | 1959 | ICPA 2043 | 1581 | ICPL 20186 | 1383 | 1482 | 32.20 |
| ICPH 2683 | 2 | 2544 | ICPA 2043 | 1581 | ICPL 99048 | 2028 | 1804 | 41.00 |
| ICPH 2695 | 2 | 4554 | ICPA 2043 | 1581 | ICPL 20099 | 1771 | 1676 | 171.73 |
| ICPH 2703 | 8 | 2711 | ICPA 2043 | 1581 | ICPL 20177 | 1862 | 1721 | 57.47 |
| ICPH 2738 | 4 | 2499 | ICPA 2047 | 1673 | ICPL 20202 | 2006 | 1839 | 35.87 |
| ICPH 2739 | 4 | 1800 | ICPA 2047 | 1673 | ICPL 20205 | 2139 | 1906 | -5.56 |
| ICPH 2743 | 2 | 2090 | ICPA 2048 | 2046 | ICPL 20202 | 2006 | 2026 | 3.17 |
| ICPH 2744 | 12 | 2036 | ICPA 2048 | 2046 | ICPL 20205 | 2139 | 2093 | -2.69 |
| ICPH 2746 | 2 | 2005 | ICPA 2048 | 2046 | ICPL 20201 | 1462 | 1754 | 14.29 |
| ICPH 2750 | 2 | 5779 | ICPA 2048 | 2046 | ICPL 99048 | 2028 | 2037 | 183.67 |
| ICPH 3331 | 4 | 2365 | ICPA 2043 | 1581 | ICPL 20186 | 1383 | 1482 | 59.60 |
| ICPH 3332 | 2 | 2019 | ICPA 2043 | 1581 | ICPL 20187 | 1381 | 1481 | 36.35 |
| ICPH 3344 | 2 | 1860 | ICPA 2047 | 1673 | ICPL 20201 | 1462 | 1567 | 18.71 |
| ICPH 3346 | 2 | 2284 | ICPA 2047 | 1673 | ICPL 20177 | 1862 | 1767 | 29.23 |
| ICPH 3349 | 6 | 1690 | ICPA 2047 | 1673 | ICPL 20186 | 1383 | 1528 | 10.63 |
| ICPH 3351 | 2 | 2192 | ICPA 2047 | 1673 | ICPL 20187 | 1381 | 1527 | 43.60 |
| ICPH 3352 | 2 | 2087 | ICPA 2047 | 1673 | ICPL 20186 | 1383 | 1528 | 36.58 |
| ICPH 3364 | 2 | 2093 | ICPA 2048 | 2046 | ICPL 20186 | 1383 | 1715 | 22.09 |
| ICPH 3462 | 2 | 1418 | ICPA 2043 | 1581 | ICPL 20096 | 1702 | 1641 | -13.57 |
| ICPH 3464 | 12 | 2064 | ICPA 2043 | 1581 | ICPL 20104 | 1813 | 1697 | 21.64 |
| ICPH 3465 | 2 | 2135 | ICPA 2043 | 1581 | ICPL 20093 | 1648 | 1614 | 32.26 |
| ICPH 3467 | 14 | 1735 | ICPA 2043 | 1581 | ICPL 20108 | 1979 | 1780 | -2.52 |
| ICPH 3476 | 6 | 1296 | ICPA 2047 | 1673 | ICPL 20096 | 2448 | 2060 | -37.10 |
| ICPH 3477 | 16 | 1832 | ICPA 2047 | 1673 | ICPL 20098 | 1806 | 1739 | 5.35 |
| ICPH 3478 | 2 | 2226 | ICPA 2047 | 1673 | ICPL 20104 | 1813 | 1743 | 27.71 |
| ICPH 3479 | 4 | 2380 | ICPA 2047 | 1673 | ICPL 20093 | 1648 | 1660 | 43.36 |
| ICPH 3481 | 4 | 1415 | ICPA 2047 | 1673 | ICPL 20108 | 1979 | 1826 | -22.50 |
| ICPH 3487 | 2 | 2829 | ICPA 2047 | 1673 | ICPL 20116 | 1822 | 1747 | 61.91 |
| ICPH 3491 | 4 | 1487 | ICPA 2048 | 2046 | ICPL 20096 | 1702 | 1874 | -20.66 |
| ICPH 3492 | 10 | 1811 | ICPA 2048 | 2046 | ICPL 20098 | 1806 | 1926 | -5.96 |
| ICPH 3494 | 16 | 1963 | ICPA 2048 | 2046 | ICPL 20093 | 1648 | 1847 | 6.26 |
| ICPH 3496 | 4 | 1137 | ICPA 2048 | 2046 | ICPL 20108 | 1979 | 2013 | -43.51 |
| ICPH 3758 | 10 | 1664 | ICPA 2092 | 1503 | ICPL 20093 | 1648 | 1575 | 5.63 |
| ICPH 3762 | 16 | 1552 | ICPA 2092 | 1503 | ICPL 20108 | 1979 | 1741 | -10.83 |
| ICPH 3803 | 2 | 1882 | ICPA 2043 | 1581 | ICPL 20102 | 1694 | 1637 | 14.94 |
| ICPH 3816 | 4 | 1468 | ICPA 2043 | 1581 | ICPL 20137 | 622 | 1101 | 33.25 |
| ICPH 3932 | 4 | 1623 | ICPA 2078 | 1094 | ICPL 20058 | 1941 | 1518 | 6.91 |
| ICPH 3934 | 2 | 1762 | ICPA 2078 | 1094 | ICPL 96053 | 1892 | 1493 | 17.99 |
| ICPH 3955 | 6 | 1822 | ICPA 2043 | 1581 | ICPL 20243 | 180 | 880 | 106.96 |
| ICPH 3958 | 6 | 1777 | ICPA 2043 | 1581 | ICPL 20238 | 335 | 958 | 85.56 |
| ICPH 3960 | 4 | 1926 | ICPA 2043 | 1581 | ICPL 83057 | 2117 | 1849 | 4.20 |
| ICPH 3963 | 2 | 2648 | ICPA 2043 | 1581 | ICPL 96053 | 1892 | 1736 | 52.51 |
| ICPH 3964 | 6 | 1452 | ICPA 2043 | 1581 | ICPL 96061 | 1672 | 1626 | -10.70 |
| ICPH 3965 | 2 | 2566 | ICPA 2043 | 1581 | ICPL 99044 | 1973 | 1777 | 44.40 |
| ICPH 3967 | 2 | 1050 | ICPA 2043 | 1581 | ICPL 99050 | 2682 | 2131 | -50.73 |
| ICPH 3970 | 2 | 1662 | ICPA 2043 | 1581 | ICPL 99055 | 2440 | 2010 | -17.34 |
| ICPH 3971 | 2 | 2290 | ICPA 2043 | 1581 | ICPL 96058 | 1892 | 1736 | 31.90 |
| ICPH 3972 | 2 | 2742 | ICPA 2043 | 1581 | ICPL 99061 | 1926 | 1753 | 56.40 |
| ICPH 4009 | 2 | 1850 | ICPA 2092 | 1503 | ICPL 20188 | 1141 | 1322 | 39.96 |
| ICPH 4012 | 12 | 1501 | ICPA 2092 | 1503 | ICPL 20186 | 1383 | 1443 | 4.03 |
| ICPH 4014 | 2 | 1775 | ICPA 2092 | 1503 | ICPL 20187 | 1381 | 1442 | 23.07 |
| ICPH 4015 | 2 | 1820 | ICPA 2092 | 1503 | ICPL 20099 | 1771 | 1637 | 11.16 |
| ICPH 4016 | 2 | 1969 | ICPA 2092 | 1503 | ICPL 20103 | 1439 | 1471 | 33.84 |
| ICPH 4022 | 10 | 1389 | ICPA 2092 | 1503 | ICPL 20116 | 1822 | 1662 | -16.47 |
| ICPH 4028 | 8 | 1772 | ICPA 2092 | 1503 | ICPL 20177 | 1862 | 1682 | 5.36 |
| ICPH 4031 | 6 | 1267 | ICPA 2092 | 1503 | ICPL 20205 | 2139 | 1821 | -30.44 |
| ICPH 4182 | 8 | 1615 | ICPA 2189 | 908 | ICPL 20108 | 1979 | 1444 | 11.88 |
| ICPH 4187 | 10 | 1750 | ICPA 2078 | 1094 | ICPL 20108 | 1979 | 1537 | 13.88 |
| ICPH 4189 | 2 | 1294 | ICPA 2078 | 1094 | ICPL 332 | 1094 | 1094 | 18.23 |
| ICPH 4193 | 2 | 1368 | ICPA 2078 | 1094 | ICPL 96061 | 1672 | 1383 | -1.12 |
| ICPH 4199 | 4 | 815 | ICPA 2078 | 1094 | ICPL 20177 | 1862 | 1478 | -44.83 |
| ICPH 4203 | 4 | 724 | ICPA 2078 | 1094 | ICPL 20243 | 180 | 637 | 13.61 |
| ICPH 4302 | 2 | 2327 | ICPA 2043 | 1581 | ICPL 20137 | 622 | 1101 | 111.34 |
| ICPH 4323 | 2 | 1562 | ICPA 2047 | 1673 | ICPL 20205 | 2139 | 1906 | -18.05 |
| ICPH 4324 | 2 | 2128 | ICPA 2047 | 1673 | ICPL 20177 | 1862 | 1767 | 20.44 |
